# The newest cathinone derivatives as designer drugs: an analytical and toxicological review

**DOI:** 10.1007/s11419-017-0385-6

**Published:** 2017-09-07

**Authors:** Milena Majchrzak, Rafał Celiński, Piotr Kuś, Teresa Kowalska, Mieczysław Sajewicz

**Affiliations:** 1 Department of General Chemistry and Chromatography, Institute of Chemistry, University of Silesia, 9 Szkolna Street, 40-006 Katowice, Poland; 2 Department of Organic Synthesis, Institute of Chemistry, University of Silesia, 9 Szkolna Street, 40-006 Katowice, Poland; 3 Toxicology Laboratory ToxLab, 6 Kossutha Street, 40-844 Katowice, Poland

**Keywords:** New synthetic cathinones, Designer drugs, NPS, LC–MS, GC–MS, NMR

## Abstract

**Purpose:**

Currently, among new psychoactive substances, cathinone derivatives constitute the biggest group, which are mainly classified into *N*-alkylated, 3,4-methylenedioxy-*N*-alkylated, *N*-pyrrolidinyl, and 3,4-methylenedioxy-*N*-pyrrolidinyl derivatives. These derivatives are actively being subjected to minor modifications at the alkyl chains or the aromatic ring to create new synthetic cathinones with the goal of circumventing laws. In this review, the new synthetic cathinones that have appeared on the illegal drug market during the period 2014–2017 are highlighted, and their characterization by gas chromatography–mass spectrometry and liquid chromatography–tandem mass spectrometry is presented.

**Methods:**

Various key words were used to conduct an extensive literature search across a number of databases, specifically for synthetic cathinones that emerged between 2014 and 2017.

**Results:**

More than 30 new cathinone derivatives were discovered. The preexisting parental compounds for the new derivatives are also referenced, and their mass spectral data are compiled in a table to facilitate their identification by forensic toxicologists.

**Conclusions:**

To our knowledge, this is the most current review presenting new synthetic cathinones. Political authorities should take measures to implement and enforce generic scheduling (comprehensive system) laws to control the diversely modified synthetic cathinones. Supplementing the existing databases with new findings can greatly facilitate the efforts of forensic toxicologists.

## Introduction

Cathinone is one of the biologically active alkaloids found in the khat shrub (*Catha edulis*). Due to its psychoactive properties, khat has been known and utilized for ages by the inhabitants of East Africa and the northeastern parts of Arabian Peninsula. In many regions, chewing of freshly collected khat leaves (thus liberating cathinone, which affects the central nervous system) is considered a matter of culture and local tradition [1–4]. Because of their structural similarity to amphetamine, cathinone and its analogs are often denoted as “natural amphetamines”, and the only structural difference between amphetamine and cathinone is the presence of a carbonyl group in the α-position of cathinone’s side chain. Similar to amphetamine, cathinone and its analogs are characterized by stimulating, euphoric, and empathogenic properties [1, 2, 4–6].

Due to their effects on the central nervous system, the first synthetic cathinone derivatives were synthesized for medicinal purposes in the early twentieth century, but they began attracting wider attention around the year 2000. At that time, synthetic cathinones were included in a broader group of psychoactive compounds denoted as “legal drugs” or “designer drugs” [5–8]. Over the course of the past 15 years, cathinone derivatives have gradually become available from so-called smart shops, through the Internet, and from drug paraphernalia stores advertising, for example, “funny items” or “aromas” [9–11]. Synthetic cathinones are most often sold as white or colored crystalline powders, and rather rarely as tablets or capsules. In the past, products containing active ingredients from the cathinone group were advertised as “plant nutrients”, “bath salts”, or “research chemicals”. Nowadays, the same substances are frequently labeled with such names as “conquerors of leeches”, “driver’s charms”, “additives to sand”, and “bidet refreshers”. Quite often, these preparations contain a combination of two or more cathinone derivatives, along with other type(s) of new psychoactive substances, caffeine, lidocaine, or benzocaine [12].

## Background

Synthetic cathinones first made their appearance in the third decade of the twentieth century, solely for medicinal purposes (to treat patients with parkinsonism, obesity, or depression), but at the beginning of the twenty-first century, they began to be consumed recreationally as substitutes for controlled drugs. After the year 2000, two pioneering representatives from this group emerged on the illegal market, namely CAT (methcathinone) and 4-MMC (mephedrone, 4-methylmethcathinone), which were followed by methylone (3,4-methylenedioxy-*N*-methylcathinone) and MDPV (3,4-methylenedioxypyrovalerone”) [6, 12, 13]. Immediately after their disclosure, the full chemical and psychoactive characteristics of these compounds were realized; as a consequence, in many countries, they became illegal, and clandestine synthetic chemists began modifying their structures to obtain new analogs. In that way, new cathinones were synthesized as substitute drugs, including butylone, ethylone, buphedrone, and an analog of the latter, pentedrone, which was soon replaced by its constitutional isomer, 4-MEC (4-methyl-*N*-ethylcathinone). About the same time, the chemical structure of mephedrone was modified by introducing new substituents to the aromatic ring; in 2009, 4-FMC (flephedrone, 4-fluoromethcathinone) and its positional isomer 3-FMC (3-fluoromethcathinone) appeared. Along with pentedrone, a second-generation synthetic cathinone, α-PVP (α-pyrrolidinopentiophenone), appeared, which belongs to the same group [5, 6, 12].

## Chemistry

The structures of the first synthetic cathinones have been continuously modified to this day, so that each year several new derivatives emerge on an illegal designer drug market. Given these circumstances, the identification of these compounds and implementation of a drug library with new structures and their physicochemical and pharmacological characteristics become an analytical challenge equally important for chemists and toxicologists.

The structures of all synthetic cathinones are derived from that of natural cathinone, and they can be considered to be phenylalkylamine derivatives, which structurally resemble the molecule of amphetamine with a carbonyl group in the α-position of the side chain adjacent to the aromatic ring. Nowadays, cathinone derivatives can be divided into four groups. Group 1 includes *N*-alkyl compounds or those with an alkyl or halogen substituent at any possible position of the aromatic ring (Table 1). The majority of the first synthetic cathinones fall into this group, and they include ethcathinone, ephedrone, mephedrone, flephedrone, buphedrone, and pentedrone. Group 2 includes methylenedioxy-substituted compounds with substituents at any given position of aromatic ring, such as methylone, pentylone, and butylone. In terms of their structure and pharmacological effect, these compounds are quite similar to 3,4-methylenedioxymethamphetamine (MDMA) (Table 2). Cathinones from group 3 (examples given in Table 3) are analogs of natural cathinone with an *N*-pyrrolidinyl substituent, and these compounds are currently the most frequently encountered in the designer drug market. Compounds which include both methylenedioxyl and *N*-pyrrolidinyl substituents belong to group 4 synthetic cathinones (Table 4) [6].

**Table 1:** Chemical names, common names, chemical structures, and molecular weights of *N*-alkylated cathinone derivatives (group 1)

Chemical name	Common name	Chemical structure	Molecular weight [Da]
[2-(*N*-Methylamino)-butan-1-onyl]-benzene	Buphedrone, α-methylaminobutyrophenone		177.24
[2-(*N*-Ethylamino)-propan-1- onyl]-benzene	Ethcathinone, ETCAT, *N*-ethylcathinone		177.24
[2-(*N*-Methylamino)-propan-1-onyl]-benzene	Ephedrone, methcathinone, CAT, α-methylaminopropiophenone		163.22
1-[2-(*N*-Methylamino)-propan-1- onyl]-4-fluorobenzene	Flephedrone, 4-FMC, 4-fluoromethcathinone		181.22
1-[2-(*N*-Methylamino)-propan-1- onyl]-4-methylbenzene	Mephedrone, 4-MMC, 4-methylmethcathinone		177.24
[2-(*N*-Methylamino)-pentan-1- onyl]-benzene	Pentedrone, α-methylaminovalerophenone		191.27
1-[2-(*N*-Methylamino)-propan-1- onyl]-3,4-dimethylbenzene	3,4-DMMC, 3,4-dimethylmethcathinone		191.27

**Table 2:** Chemical names, common names, chemical structures, and molecular weights of 3,4-methylenedioxy-*N*-alkylated cathinone derivatives (group 2)

Chemical name	Common name	Chemical structure	Molecular weight [Da]
1-[2-(*N*-Methylamino)-butan-1-onyl]-(3,4-methylenedioxy)-benzene	Butylone, bk-MBDB, β-keto-methylbenzodioxolylbutanamine		221.25
1-[2-(*N*-Ethylamino)-propan-1-onyl]-(3,4-methylenedioxy)-benzene	Ethylone, bk-MDEA, 3,4-methylenedioxy-*N*-ethylcathinone		221.25
1-[2-(*N*-Methylamino)-propan-1-onyl]-(3,4-methylenedioxy)-benzene	Methylone, bk-MDMA, 3,4-methylenedioxy-*N*-methylcathinone		207.23
1-[2-(*N*-Methylamino)-pentan-1-onyl]-(3,4-methylenedioxy)-benzene	Pentylone, bk-MBDP		235.28

**Table 3:** Chemical names, common names, chemical structures, and molecular weights of *N*-pyrrolidine cathinone derivatives (group 3)

Chemical name	Common name	Chemical structure	Molecular weight [Da]
1-[2-(Pyrrolidin-1-yl)-hexan-1-onyl]-4-methylbenzene	MPHP, 4-methyl-α-pyrrolidinohexanophenone		259.39
1-[2-(Pyrrolidin-1-yl)-pentan-1-onyl]-benzene	α-PVP, α-pyrrolidinovalerophenone		231.33
1-[2-(Pyrrolidin-1-yl)-pentan-1-onyl]-4-methylbenzene	Pyrovalerone, 4-methyl-α-pyrrolidinovalerophenone		245.36

**Table 4:** Chemical names, common names, chemical structures, and molecular weights of 3,4-methylenedioxy-*N*-pyrrolidine cathinone derivatives (group 4)

Chemical name	Common name	Chemical structure	Molecular weight [Da]
1-[2-(Pyrrolidin-1-yl)-butan-1-onyl]-3,4-methylenedioxybenzene	MDPBP, 3,4-methylenedioxy-α-pyrrolidinobutiophenone		261.32
1-[2-(Pyrrolidin-1-yl)-propan-1-onyl]-3,4-methylenedioxybenzene	MDPPP, 3,4-methylenedioxy-α-pyrrolidinopropiophenone		247.29
1-[2-(Pyrrolidin-1-yl)-pentan-1-onyl]-3,4-methylenedioxybenzene	MDPV, 3,4-methylenedioxypyrovalerone		275.34

## Mechanisms of action and metabolism

**Fig. 1:**
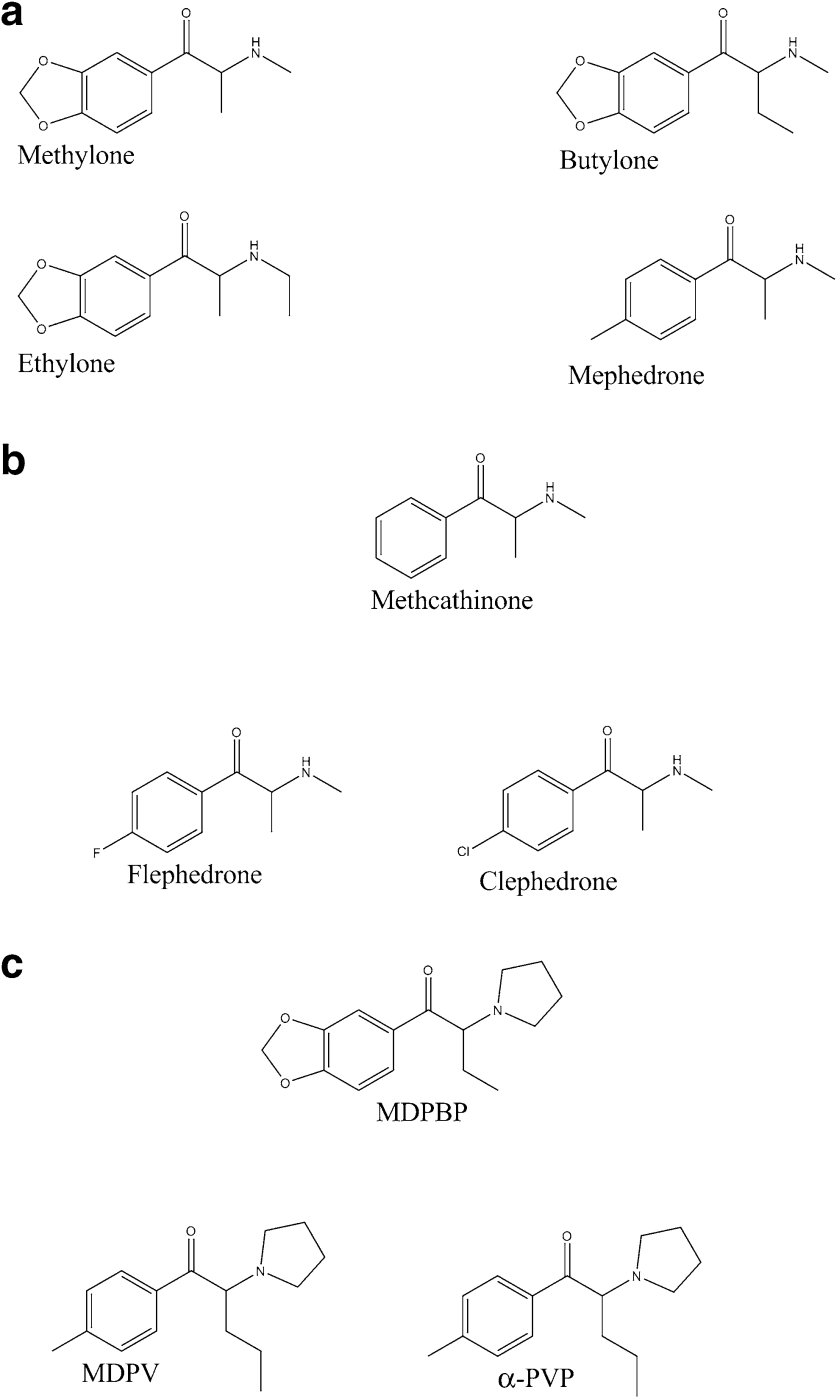
Chemical structures of **a** cocaine-MDMA-mixed cathinones, **b** methamphetamine-like cathinones, and **c** pyrovalerone-cathinones as a function of their in vitro pharmacological activities

In vitro experiments have shown that synthetic cathinones easily penetrate the blood-brain barrier (BBB) [13]. Cathinone and its derivatives (denoted as β-keto-amphetamines) exert a stimulating and sympathomimetic effects on the central nervous system due to an increased concentrations of catecholamines in the inter-synapse spaces, and their effects are generally much stronger than that of amphetamine itself [2, 14–21]. Similar to amphetamine, cathinones exist as two stereoisomeric forms, and each is characterized by different potency [6]. The mechanism of action of synthetic cathinones involves the inhibition of monoamine transporters such as dopamine transporter (DAT), noradrenaline transporter (NAT), and serotonin transporter (SERT). Depending on the derivative, and more precisely, on its chemical structure, their affinity to the aforementioned transporters can be different. Differential selectivity toward individual monoamines differentiates the synthetic cathinones in terms of their effects on neurotransmission [14, 17, 21]. Considering two properties of synthetic cathinones, i.e., the potency of their inhibition of dopamine, noradrenaline and serotonin reuptake as well as their ability to liberate these compounds, Simmler et al. [13] classified them into three groups on the basis of in vitro experiments. The first group includes cathinones that act in a way similar to cocaine and MDMA, and it is denoted as the "*cocaine*-*MDMA*-*mixed cathinone*” group. The mechanism of action of the cathinones belonging to this group involves rather non-selective inhibition of monoamine reuptake (in that way resembling cocaine, which shows greater selectivity toward the dopamine transporter than the serotonin transporter) and promotion of serotonin liberation (similar to MDMA). Substances belonging to this group which exhibit action similar to cocaine include mephedrone, methylone, ethylone, and butylone, whereas naphyrone acts similarly to MDMA [13, 14, 16–18, 20]. The second group includes cathinones that act similarly to methamphetamine, and its representatives are denoted as “*methamphetamine*-*like cathinones*”. Their mechanism of action involves preferential reuptake inhibition of catecholamines and liberation of dopamine, and the representatives of this group are methcathinone, flephedrone, and clephedrone (4-chloromethcathinone) [13, 14]. The third pharmacological effect on neurotransmission is induced by synthetic cathinones with structures based on that of pyrovalerone, and these compounds are therefore denoted as “*pyrovalerone*-*cathinones*”. The representatives of the third group are MDPV and MDPBP, recognized as very potent and selective inhibitors of the catecholamine reuptake, which do not demonstrate the neurotransmitter liberating effect [13, 15]. A cathinone classification scheme based on their interaction with cathecholamines is given in Fig. 1.

Reaction symptoms of human organisms following an intake of the discussed cathinone derivatives conform to the aforementioned mechanism of action for the individual cathinone groups, as revealed in the in vitro experiments and defined on the basis of neurotransmission level [17]. The extent and strength of cathinone action on the central nervous system can be very broad, and they depend on such factors, as age, sex, degree of addiction, general health condition, use of medication, an intake of other hallucinogenic or psychotropic agents, and use of alcohol. However, the subjective feelings of cathinone users are rather similar and are said to involve strong excitation, euphoria, increased empathy, increased self-assurance and interpersonal openness, and increased libido [2, 5, 6, 12]. It must be clearly stated that both chronic exposure to the action of synthetic cathinones and a single or sporadic intake can be equally hazardous to human health and life. Among discomforts experienced by the consumers of “bath salts” and similar products, vomiting, sweating, short-term memory problems, migraines, feeling giddy, excessive heart rate, and muscle tremors are most common. Neurologically, cathinone overdose can result in memory disturbances, memory loss, fits of panic and aggression, hallucinations, depression, and even fits of psychoses with suicidal thoughts [13]. From the standpoint of cardiology, synthetic cathinones evoke elevated blood pressure, heart arrhythmia, tachycardia, and cardiac arrest. Among the more frequent effects of cathinone use are hyponatremia, hyperthermia, anemia, and rhabdomyolysis [16].

The metabolism of synthetic cathinones is relatively well known. The metabolism of mephedrone presented by Meyer et al. [18] is understood to involve *N*-demethylation to basic amines as the main mode of decomposition, followed by reduction of the ketone functionality to 4-methylnorephedrine and hydroxylation of the methyl substituent of the aromatic ring, which gives rise to its final oxidation to the respective carboxylic acid. Uralets et al. [22] studied the metabolites of 16 synthetic cathinones found in human urine upon dividing them into three groups of cathinone derivatives. The first group included mephedrone, buphedrone, 4-methylbuphedrone, pentedrone, 4-methylethcathinone (4-MEC), 3,4-DMMC, *N*-ethylbuphedrone, flephedrone, and ethcathinone, which were metabolized following the pattern of the synthetic cathinone precursors (i.e., methcathinone and cathinone). In the urine of people treated with these compounds, metabolites resulting from β-ketone reduction and *N*-dealkylation were detected—norephedrines and ephedrines as the main metabolites. The second group included 3,4-methylenedioxy-substituted cathinones (i.e., methylone, butylone, and ethylone), which exhibited less effective β-keto reduction than the compounds from the first group, which might be due to the presence of the 3,4-methylenedioxyl substituent in the aromatic ring. Thus in the urine analyzed, the parent molecules were detected. The third group included α-pyrrolidinophenones such as α-PVP and α-PBP, which were thought not further metabolized following reduction of the ketone group, or were found to be structurally unchanged in the urine [18, 22, 23]. However, Shima et al. [24] showed that the main metabolic pathways of α-pyrrolidinophenones change significantly depending on the alkyl chain length of the parent molecule in humans. The metabolism of PV9 differed remarkably from that of α-PVP and α-PBP. The metabolic pathways of PV9 involved reduction of the ketone group to the corresponding alcohol, oxidation of the pyrrolidine ring to the corresponding pyrrolidone, aliphatic oxidation of the terminal carbon atom to the corresponding carboxylate, and hydroxylation at the penultimate carbon atom to the corresponding alcohol, followed by further oxidation to the ketone, and combinations of these steps [24].

## Case reports on intoxication with cathinones

The first reported death from mephedrone, one of the earliest available and most popular designer drugs on the market, occurred in 2008 in Sweden [25]. Since then, 45 mephedrone-induced deaths have been reported in England, 12 fatal cases in Scotland, one each in Wales and North Ireland, and one in Guernsey Island [26]. By August 2011, 90 documented cases of death related to mephedrone intoxication had been reported in Great Britain. The majority of the victims were young males between 25 and 34 years of age, with documented past histories of drug abuse [26]. In 2013, Adamowicz et al. [27] described a case of fatal intoxication caused by mephedrone in Poland. The first documented case of fatal overdose with MDPV occurred in the USA in 2012, and was followed by two similar deaths (20-year-old male and 48-year-old female) [28]. Methylone and MDPV (together with mephedrone) have caused the death of several individuals between the age of 19–38 during a similar time period [29]. An expanding library of synthetic cathinones has resulted in the intake of new brand compounds, with potencies that are unprecedented, even among drug users. Quite often, drug consumers are not even aware of which psychoactive compounds are present in the product that they are going to take [30]. Most recently, the first death caused by the newest generation of synthetic cathinones available on the synthetic drug market was reported by Hasegawa et al. [31]; they described a case of PV9 intoxication involving an 18-year-old female who developed convulsions and muscle trembling, and who finally lost consciousness upon intake of an “aroma liquid”. Despite nearly 20 h of intense medical rescue efforts (which consisted of gastric lavage and intravenous transfusions), the girl eventually died. Nine tissues were collected from her body in the course of autopsy, i.e., skeletal muscle, pancreas, fat tissue, kidney, liver, lung, spleen, heart muscle, and brain. The highest concentration of PV9 (1-phenyl-2-(pyrrolidin-1-yl)octan-1-one) was detected in the kidney, which suggested fast removal of this compound via the urine. The first multiple drug intoxication caused by the simultaneous ingestion of three cathinone derivatives (i.e., 4-methoxy-PV8, PV9, and 4-methoxy-PV9) was reported by Kudo et al. [32]; a woman in her 30s was found dead after consuming alcohol, and the “aroma drugs” and “bath salts” were found next to her body.

## Extraction methods

From an analytical point of view, the proper preparation of samples for instrumental analysis is a crucial and decisive step. This section deals with the analysis of biological material (blood, urine, or body tissues), which is usually a challenging task due to the possible presence of a vast number of metabolites and other biochemical components of the samples, which can jointly generate considerable measurement errors. Only the adequate extraction of a biological matrix allows an accurate quantification of toxic compounds while preventing the contamination of sensitive analytical instruments with impurities. The most frequently applied techniques for the isolation of psychoactive and hallucinogenic compounds from biological matrices are liquid-liquid extraction (LLE) and solid-phase extraction (SPE). Detailed working parameters for these techniques depend on the type of tissue analyzed and on the chemical nature of the substance of interest (mainly, on its acidity or basicity). In the paper by Dickson et al. [33], the following method for preparation of the autopsy material was described for basic drug screening: to 1 or 2 mL liquid sample, ca. 3 mL 0.1 M phosphate buffer (pH 6) and an internal standard (mepivacaine or ethylmorphine at the concentration of 0.5 mg L^−1^) were added, and then the thus prepared mixture was ultrasonicated for 15 min and centrifuged. The prepared samples were applied to the top of the SPE cartridges (mixed-mode silica-based SPE, ZCDAU020) which were previously conditioned with 3 mL methanol, 3 mL deionized water, and 2 mL of the same phosphate buffer. After that, the cartridges were rinsed with 2 mL deionized water, 2 mL 20% aqueous acetonitrile, and 2 mL 0.1 M acetic acid. Finally, the cartridges were dried for 3 min in vacuum, ultimately rinsed with 2 mL hexane and 3 mL methanol, and once again dried for 10 min in vacuum. The adsorbed analytes were then eluted with 3 mL dichloromethane/isopropanol/ammonium hydroxide (78:20:2, v/v/v), and after evaporation of solvent under a stream of nitrogen and dissolution of the residue in 50 µL acetonitrile, the samples were ready for instrumental analysis. The analogous SPE procedures were applied by other toxicological analysts during their investigations of intoxications involving cathinone derivatives [34, 35].The innovative introduction of the QuEChERS technique for toxicological analysis should be mentioned in most up-to-the-date reports on the identification of synthetic cathinones from postmortem samples. This quick (Qu), easy (E), cheap (Ch), effective (E), rugged (R) and safe (S) approach was initially introduced for the quantification of pesticide contaminants in food. Application of this approach to toxicological analysis was motivated by the fact that the LLE and SPE approaches are subject to the possible contamination of samples by unwanted impurities that give rise to inaccurate final results and negative matrix effects on sensitive analytical instruments. Thus, in 2012, Usui et al. [36] applied the QuEChERS method for rapid extraction of psychoactive substances from human blood, which demonstrated selectivity comparable to SPE, and was as simple as LLE. QuEChERS was also much faster and cheaper than both LLE and SPE. This was a two-step procedure. In the first step (denoted as *extraction/partitioning*), liquid samples (e.g., blood) undergo triple dilution with distilled water, and then they are placed in plastic test tubes that contain 0.5 g of a commercial preparation composed of magnesium sulfate and sodium acetate, a stainless steel bead, and 1 mL acetonitrile containing IS. The contents of the test tube are intensely mixed and centrifuged. With acidic analytes, the obtained acetonitrile layer can be subjected directly to instrumental analysis. To extract basic compounds, the second step must also be performed, i.e., the “*dispersive solid phase extraction (dSPE)”*, which involves introducing 600 µL acetonitrile supernatant to a test tube containing a commercial mixture of *N*-propylethylenediamine (primary secondary amine, PSA), a portion of an end-capped octadecylsilane, and magnesium sulfate, for purification. Then the test tube contents are mixed and centrifuged, and an upper layer is subjected to instrumental analysis. Because of the advantages of the this method (i.e., its speed, lower risk of the instrument contamination, and general cost-effectiveness), it is now the most frequently used analytical approach (either directly or with minor modifications to the procedure) for toxicological analysis of biological samples [36, 37].

## Detection techniques

The steadily growing market of designer drugs poses a permanent analytical challenge for those who focus on the physicochemical characterization of drugs and their identification in biological samples. Apart from the key stage of sample preparation, a crucial role is played by analytical techniques used to assess the chemical composition of these drugs. Considerable progress in chromatographic and spectroscopic techniques (resulting in sophisticated instruments able to identify hundreds of compounds in nanomole concentrations) allows the scope of toxicological investigations to be expanded from the currently searched—both known and unknown—designer drug molecules to their metabolites [38, 39]. Each of these two analytical techniques has praiseworthy advantages, and both carry certain drawbacks at the same time; however, the combination of the two methods provides a powerful tool for the identification and quantification of cathinones in products and in biological samples [38–43].

Practically all attempts to identify psychoactive compounds (the cathinone derivatives included) begin with the application of the non-specific screening methods. In the case of product samples (e.g., powders, tablets and the contents of capsules), standard colorimetric methods are used, and they constitute a routine service in most analytical institutions, including police forensic laboratories [43, 44]. The most common test for compounds which contain a nitrogen atom (widely used for identification of amphetamine) makes use of the Marquis reagent (sulfuric acid and formaldehyde). It does not give rise to a color reaction with synthetic cathinones derived from mephedrone, but it gives positive results with the compounds containing the methylenedioxyl substituent, such as MDPV. For this latter cathinone, an additional test with the Chen reagent (acetic acid, copper monosulfide, and sodium hydroxide) can also be applied, and this test is suitable for the ephedrine derivatives as well [44]. Colorimetric techniques are advantageous in the sense that they are rapid and easy to apply. However, they usually only allow identification of a single structural fragment of a given molecule, which is not a sufficient criterion for designating a compound to a given group of derivatives. Because of this shortcoming, the identification of synthetic cathinones cannot be carried out via the use of colorimetric methods; they are generally not used for the preliminary screening of designer drugs.

The screening of biological material is most often carried out through the use of immunoenzymatic assays. The most common assay is ELISA (enzyme-linked immunosorbent assay), which is devised to detect certain psychoactive substances in an investigated sample via mono- or polyclonal antibodies coupled with an enzyme. This technique is very popular in biomedical analysis, e.g., in virology (the HIV test) and bacteriology (the mycobacterium test) and in food analysis targeting potential allergens [45]. In most commercial laboratories, immunoenzymatic assays for toxicological purposes are also applied. It can be used as a screening technique for the detection of synthetic cathinones in biological samples [46, 47], but it is considered to be non-specific due to possible cross reactions, such as the reaction between MDPV and butylone [47].

Screening analyses can be used as a preliminary step in an assessment of product samples and/or biological samples for the presence of psychoactive compounds. Their results often indicate a point of focus for further investigations with a narrower group of suspected compounds, but a decisive role is played by specific analytical techniques. For synthetic cathinones, the analytical techniques of first choice are gas chromatography (GC) and liquid chromatography (LC) coupled with different spectroscopic instruments.

Gas chromatography–mass spectrometry (GC–MS) is the most frequently used instrumental technique for toxicological analysis. It is applicable to many volatile psychoactive compounds (cathinones included) [35, 37, 43, 48–52]. Moreover, the time of a single analytical run is relatively short and within the period of ca. 40 min; a vast number of compounds can be screened in this way [48]. During GC–MS, chemical ionization (CI) is occasionally applied [48], but in most cases, the electron-ionization (EI) mode predominates [35, 37, 43, 48–52]. The cathinone mass spectrum originating from GC–MS in the positive ionization mode is very simple and characterized by signals derived from the iminium ions. However, identification of different cathinone derivatives becomes rather complicated [35, 48–52]. For this reason, different modifications of the detection are highly valuable. Tandem mass spectrometry (MS/MS) constitutes one such method which provides more information about the molecular structure and greatly facilitates identification. In 2012, Zuba [48] proposed a novel analytical procedure for the determination of synthetic cathinones by means of GC–EI-MS. According to his approach, if the iminium ion appears as an intense ion (*m/z* = 16 + 14*n*, where *n* = 1, 2, 3 etc.), it can be assumed that the cathinone present in the analyzed sample is characterized by having a straight aliphatic chain. If in the mass spectrum a signal appears which corresponds to the pyrrolidine ion (*m/z* = 70 + 14*n*, where *n* = 1, 2, 3 etc.), then the identified cathinone should contain a pyrrolidine ring. Due to the possible existence of many different regioisomers for different cathinone derivatives, the assessment of the aliphatic chain length and its possible substituents and identification of the substituents on the aromatic ring define essential tasks. With unsubstituted rings, the presence of the fragmentation ions at *m/z* 77 and/or 105 is characteristic. Signals at *m/z* 91 and/or 119 are characteristic of the methylphenyl ring, and those at *m/z* 121 and/or 149 suggest the presence of the methylenedioxyl ring substituent. The GC–EI-MS technique is rapid, yet its main drawbacks include the possible emergence of identical fragmentation patterns for certain isomers and the low intensity of molecular ions when applying the EI mode. These are the main reasons why the application of alternative mass spectrometric techniques often becomes inevitable [48].

However, recently, the effective use of GC–EI-MS/MS for distinguishing certain regioisomers of cathinone derivatives has been reported in the literature [49]. An effective method for fragmentation of iminium ions has been described, which makes it possible to clearly distinguish cathinone derivatives with the same aminoalkyl moiety. For example, in the case of pentedrone, *N*-ethylbuphedrone, 4-methyl-*N*-dimethylbuphedrone, and *N*-ethylmethcathinone, it has been shown that secondary and tertiary fragmentation of the iminium ion is useful for the differentiation of the above four compounds; the compounds can be discriminated by GC–MS/MS based on the intensity difference of the product ions originating from the iminium ion. This work has proposed that various cathinones and forthcoming novel illicit drugs can be differentiated based on a detailed analysis of product ion spectra derived from the iminium and acylium ions, if combined with an LC-photodiode array (PDA) analysis [49].

Liquid chromatography–mass spectrometry (LC–MS) is employed in toxicological analysis laboratories nearly as frequently as GC–MS, and enjoys high popularity due to its high sensitivity and selectivity [38, 39, 53]. Most LC–MS analyses are carried out in the tandem MS mode with the multiple reaction monitoring (MRM) or the selected reaction monitoring (SRM) mode, and the most frequently used ionization interface is electrospray ionization (ESI) [52–54]. In the case of synthetic cathinones, a vast number of analyses have been carried out in the ESI-MS^n^ mode, which has enabled observation of product ion formation patterns characteristic of the respective protonated molecular ions. Characteristic features of the product ion formation include the loss of a water molecule and the split-off of the pyrrolidine ring [53]. Lesiak et al. [42] presented a different type of analysis in the context of a mixture of cathinone derivatives present in a commercial product labeled as a “bath salt”. These authors admitted that the most popular and most frequently applied analytical techniques were GC–MS and LC–MS supported by libraries of mass spectra, but the utility of these approaches is declining in the face of the avalanche of novel cathinone derivatives that appear with increasing frequency on the designer drug market. As an alternative, these authors proposed application of the DART (direct analysis in real time) ionization source coupled with the mass spectrometer. The results obtained with this approach pointed to its greater utility and enhanced capacity to differentiate compounds that are structurally closely related or even isomeric, both as individual species and as the components of mixtures. Ultra-high-performance liquid chromatography (UHPLC) coupled with time-of-flight mass spectrometry (TOF-MS) and its quadrupole TOF (QTOF) modification is an additional technique which can be used for analysis of the active ingredients in designer drugs. Using these latter techniques, Ibáñez et al. [41] successfully identified compounds (including certain cathinone derivatives) present in numerous designer drugs commercialized as tablets, capsules, powders, and dried herbs. The results obtained by these authors demonstrated the high potential of the discussed techniques in applications toward both “target analysis” and “non-target analysis” of psychoactive compounds, where each newly emerging compound was regarded as an unknown substance. An advantage of the QTOF-MS-based methods is that preliminary identification of the analyzed compounds can be performed without any reference standards; at the initial stage of investigations they are not needed. Reference standards are only acquired at the final stage of investigations to ultimately confirm the presence of a given compound, once solid instrumental evidence is already at hand.

A less frequently employed detection system, also applied during the investigation of drug products and biological samples, is LC coupled with ultraviolet-visible (UV-Vis) spectroscopy using diode array or PDA detection [53, 55–58]. With this detection system, one can record the UV-Vis spectra of the investigated cathinones and establish the absorption wavelength characteristics of the individual group representatives. These data can be added to a library, thus providing physicochemical characteristics of individual cathinone species.

Last but not least, in the context of the structural elucidation of cathinone derivatives, during the analysis of the evidence material, the nuclear magnetic resonance (NMR) and infrared absorption spectroscopy cannot be forgotten [40, 52, 53]. By way of NMR spectroscopy, the substitutional isomerism of a given molecule can be defined without using a reference standard. Obviously, this technique cannot be employed to quantify the contents of psychoactive substances in biological material, but ^1^H and ^13^C NMR spectroscopy are commonly used for detailed assessment of the chemical structures of cathinone derivatives, including their substitutional isomerism [40, 52, 53, 59].

## Most recently described derivatives and their characterization

Due to the wide-ranging possibility for structural modification of cathinones, new compounds from this group are continuously emerging on the global designer drug market, and thus their identification and physicochemical characterization pose a serious analytical challenge. The exchange of information on new derivatives, including the full physicochemical characteristics of these compounds, and proposals for methods that are uniquely suited for their identification, combined with reports on cases of intoxication, contribute to the dynamically developing field of toxicological analysis.

Beginning in early 2000s, when synthetic cathinones first appeared on the market [6], new derivatives have been continuously reported in the literature [8, 9, 11]. In the past 2 years, over a dozen new cathinone derivatives have been commercialized [9]. In mid-2013, a methoxy derivative was identified for an already known synthetic cathinone, α-PVP (i.e., 4-methoxy-α-PVP [56]), in a product which also contained 4-methylbuphedrone. Then, in March of 2014, Uchiyama et al. [55] reported as many as seven new synthetic cathinones. The authors analyzed multicolored liquids marketed as “aroma liquids” and colored powders advertised as “fragrance powders”. First, all of these liquids and powders were subject to liquid extraction, after which 2-mg aliquots of powders and 20-µL aliquots of liquids were extracted with 1 mL methanol through the use of ultrasonication. After centrifugation and filtering, if necessary, the obtained supernatants were diluted and subsequently analyzed by ultra-high-performance liquid chromatograph coupled with ESI-MS and GC–EI-MS. The exact molar mass (Da)-to-charge (*z*) ratios of the compounds of interest (*m*/*z*) were measured by LC–QTOF-MS. Additionally, the structures of all of these new derivatives were confirmed by means of ^1^H and ^13^C NMR spectroscopy. This study resulted in the identification of seven new cathinones, i.e., MPHP (4-methyl-α-pyrrolidinohexanophenone), α-PHPP (α-pyrrolidinoheptanophenone, PV8), α-POP (α-pyrrolidinooctanophenone, PV9), 3,4-dimethoxy-α-PVP (3,4-dimethoxy-α-pyrrolidinopentiophenone), 4-F-α-PVP (4-fluoro-α-pyrrolidinopentiophenone), α-EAPP (α-ethylaminopentiophenone), and *N*-ethyl-4-methylpentedrone (4-methyl-α-ethylaminopentiophenone). Less than a half year later, and using the same extraction and analytical approach, Uchiyama et al. [57] added four newly identified compounds to the database of synthetic cathinones, i.e., α-PHP (α-pyrrolidinohexanophenone), 4-methoxy-α-POP (4-methoxy-α-pyrrolidinooctanophenone), 4-methoxy-α-PHPP (4-methoxy-α-pyrrolidinoheptanophenone), and 4-F-α-PHPP (4-fluoro-α-pyrrolidinoheptanophenone). In addition, a growing tendency was noted for the delivery of mixtures of designer drugs in commercialized products. These were usually binary and ternary mixtures and not necessarily of a single class of compounds, but instead consisted of drugs belonging to different groups (e.g., as combinations of synthetic cathinones and synthetic cannabinoids) [55–57]. Due to the unknown mechanism of action and toxicity of these new psychoactive compounds, their combination can result in an unexpected synergism and consequently jeopardize the health and life of the potential drug takers.

In the second half of 2015, Doi et al. [60] reported for the first time the discovery of thienyl cathinone derivatives in commercialized designer drugs, including α-PBT (α-pyrrolidinobutiothiophenone), and in addition, the bromothienyl analogs of α-PVT (α-pyrrolidinopentiothiophenone) and α-PBT. At approximately the same time, Gambaro et al. [54] reported on a new cathinone derivative, thiothinone [(2-methylamino)-1-(2-thienyl)-1-propanone].

Complementary information and an expansion of the physicochemical database regarding α-PHP, along with the first report on a new cathinone derivative, 4-fluoro-PV9 (4-fluoro-α-pyrrolidinooctanophenone), were provided by our research group at the end of 2015 [53]. Apart from standard applications of LC–MS, GC–MS, and NMR, the authors characterized these two compounds through the use of MS/MS with electrospray ionization (ESI-MS^n^), Fourier transform infrared spectroscopy, differential scanning calorimetry, and thermogravimetric analysis. Moreover, the analyzed materials (multicolored powders) were not extracted with methanol; instead they were treated with in-home elaborated solvent systems. In the first stage, 10 mg of a given powder was dissolved in 1 mL acetonitrile/methanol (50:50, v/v), then ultrasonicated and centrifuged, and the obtained supernatant was dissolved in methanol/water (80:20, v/v) for instrumental analysis [53].

Full characterization of nine new cathinones—*N*-ethylhexedrone, 4-Cl-pentedrone, 4-Cl-EAPP, propylone, *N*- ethylnorpentylone, 6-methoxy-bk-MDMA, α-PiHP, 4-Cl-α-PHP, and 4-F-α-PHP—was described by Liu et al. [61].

This year, Błażewicz et al. [62] described four novel synthetic cathinones—hexedrone, 4-BEC, 4-Cl-PPP, and 4-Br-PVP [62]. In May of this year, three new cathinone derivatives—4-MPD, 4-F-α-PHP, and bk-EPDP—were also described [63].

In Table 5, the names and structures of recently reported cathinone derivatives are shown according to the featured structures.

**Table 5:** Chemical names, common names, and chemical structures of the most recently reported cathinone derivatives, along with respective reference sources, arranged according to the featured structures

Chemical name	Common name	Chemical structure	Publication year and reference
2-Methylamino-1-(phenyl)hexan-1-one	Hexedrone, HEX		2017 [62]
1-(4-Chlorophenyl)-2-(methylamino)-pentan-1-one	4-Cl-pentedrone		2017 [61]
1-(4-Methylphenyl)-2-(methylamino)-pentanone	4-Methylpentedrone, 4-MPD		2017 [63]
1-[2-(*N*-Ethylamino)-pentan-1- onyl]-benzene	α-EAPP, α-ethylaminopentiophenone		2014 [55]
2-(Ethylamino)-1-phenylhexan-1-one	*N*-Ethylhexedrone		2017 [61]
1-(4-Chlorophenyl)-2-(ethylamino)pentan-1-one	4-Cl-EAPP		2017 [61]
1-(4-Bromophenyl)-2-(ethylamino)propan-1-one	4-Bromoethcathinone, 4-BEC		2017 [62]
1-[2-(*N*-Ethylamino)-pentan-1- onyl]-4-methylbenzene	*N*-Ethyl-4-methylpentedrone, 4-methyl-α-ethylaminopentiophenone		2014 [55]
1-(3,4-Methylenedioxyphenyl)-2-ethylaminopentan-1-one	*N*-Ethylnorpentylone, bk-EPDP, *N*-ethylpentylone		2017 [61, 63]
1-(3,4-Methylenedioxyphenyl)-2-propylaminopropan-1-one	Propylone		2017 [61]
1-(6-Methoxy-3,4-methylenedioxyphenyl)-2-methylaminopropan-1-one	6-Methoxy-bk-MDMA		2017 [61]
1-[2-(Pyrrolidin-1-yl)-hexan-1- onyl]-benzene	α-PHP, α-pyrrolidinohexanophenone		2014 [57]
4-Methyl-1-phenyl-2-(pyrrolidin-1-yl)pentan-1-one	α-PiHP		2017 [61]
1-[2-(Pyrrolidin-1-yl)-heptan-1- onyl]-benzene	α-PHPP, PV8, α-pyrrolidinoheptanophenone		2014 [55]
1-[2-(Pyrrolidin-1-yl)-octan-1- onyl]-benzene	α-POP, PV9, α-pyrrolidino-octanophenone		2014 [55]
1-[2-(Pyrrolidin-1-yl)-pentan-1-onyl]-4-fluorobenzene	4-F-α-PVP, 4-fluoro-α-pyrrolidinopentiophenone		2014 [55]
1-(4-Fluophenyl)-2-(pyrrolidin-1-yl)hexan-1-one	4-F-α-PHP		2017 [61]
1-[2-(Pyrrolidin-1-yl)-heptan-1- onyl]-4-fluorobenzene	4-F-α-PHPP, 4-fluoro-α-pyrrolidinoheptanophenone		2014 [57]
1-[2-(Pyrrolidin-1-yl)-octan-1- onyl]-4-fluorobenzene	4-F-α-PV9, 4-fluoro-α-POP, 4-fluoro-α-pyrrolidinooctanophenone		2016 [53]
1-(4-Chlorophenyl)-2-(pyrrolidin-1-yl)propan-1-one	4-Cl-α-PPP		2017 [62]
1-(4-Chlorophenyl)-2-(pyrrolidin-1-yl)hexan-1-one	4-Cl-α-PHP		2017 [61]
1-(4-Bromophenyl)-2-(pyrrolidin-1-yl)pentan-1-one	4-Br-α-PVP		2017 [62]
1-[2-(Pyrrolidin-1-yl)-pentan-1-onyl]-4-methoxybenzene	4-Methoxy-α-PVP, 4-methoxy-α-pyrrolidinopentiophenone		2014 [56]
1-[2-(Pyrrolidin-1-yl)-heptan-1-onyl]-4-methoxybenzene	4-Methoxy-α-PHPP, 4-methoxy-α-pyrrolidinoheptanophenone		2014 [57]
1-[2-(Pyrrolidin-1-yl)-octan-1- onyl]-4-methoxybenzene	4-Methoxy-α-POP, 4-methoxy-α-pyrrolidinooctanophenone		2014 [57]
1-[2-(Pyrrolidin-1-yl)-pentan-1-onyl]-3,4-dimethoxybenzene	3,4-Dimethoxy-α-PVP, 3,4-dimethoxy-α-pyrrolidinopentiophenone		2014 [55]
2-(Methylamino)-1-(2-thienyl)-1-propanone	Thiothinone		2016 [54]
2-(Pyrrolidin-1-yl)-1-(thiophen-2-yl)butan-1-one	α-PBT		2016 [60]
1-(5-Bromothiophen-2-yl)-2-(pyrrolidin-1-yl)butan-1-one	5-Br-α-PBT		2016 [60]
	*x* = Br		
	*y* = *z *=* H*		
1-(4-Bromothiophen-2-yl)-2-(pyrrolidin-1-yl)butan-1-one	4-Br-α-PBT		
	*y* = Br		
	*x* = *z *=* H*		
1-(3-Bromothiophen-2-yl)-2-(pyrrolidin-1-yl)butan-1-one	3-Br-α-PBT		
	*z* = Br		
	*x* = *y* = *H*		
1-(5-Bromothiophen-2-yl)-2-(pyrrolidin-1-yl)pentan-1-one	5-Br-α-PVT		2016 [60]
	*x* = Br		
	*y* = *z *= *H*		
1-(4-Bromothiophen-2-yl)-2-(pyrrolidin-1-yl)pentan-1-one	4-Br-α-PVT		
	*y* = Br		
	*x* = *z* =* H*		
1-(3-Bromothiophen-2-yl)-2-(pyrrolidin-1-yl)pentan-1-one	3-Br-α-PVT		
	*z* = Br		
	*x* = *y *= *H*		
1-(4,5-Bromothiophen-2-yl)-2-(pyrrolidin-1-yl)pentan-1-one	4,5-Br-α-PVT		
	*x* = *y* = Br		
	*z* = *H*		

In Table 6, a summary of analytical data for the recently reported cathinone derivatives is listed also according to the featured structures.

**Table 6:** Common names, absorption maxima, molecular weights, liquid chromatography-electrospray ionization-tandem mass spectrometry (LC–ESI-MS/MS) peaks, gas chromatography–electron ionization-mass spectrometry (GC–EI-MS) peaks, and references for the most recently reported cathinone derivatives, arranged according to the featured structures

Common name	Absorption maxima [nm]	Molecular weight [Da]	Precursor ion [M + H^+^] and product ions by LC–ESI-MS/MS [*m/z*]	Base peak and other peaks of GC–EI-MS spectrum [*m/z*]	Reference
Hexedrone, HEX	224	205.15	**206**, 188, 132, 175, 100, 105, 119, 91	**100**, 77, 69, 44, 58	[62]
4-Cl-pentedrone	No data	225.10	**226**, 144, 208, 125, 166, 164, 178, 173, 131, 138	**86**, 44	[61]
4-Methylpentedrone, 4-MPD	–	205.15	**206**, 188, 158, 146, 145, 144, 131, 130	**86**	[63]
α-EAPP	251	205.30	**206**	**100**, 77	[55]
*N*-Ethylhexedrone	No data	219.17	**220**, 130, 202, 146, 91, 158, 175	**114**, 105, 58	[61]
4-Cl-EAPP	No data	239.12	**240**, 158, 164, 125, 180, 138, 187, 145, 195, 192	**100**, 58	[61]
4-Bromoethcathinone, 4-BEC	265	255.03	**256**/**258**, 159, 144, 132	**72**, 44, 185, 155	[62]
*N*-Ethyl-4-methylpentedrone	264	219.32	**220**	**100**, 91	[55]
*N*-Ethylnorpentylone, bk-EPDP, *N*-ethylpentylone	No data	249.14	**250**, 202, 189, 232, 175, 203, 149, 173	**100**, 149, 91, 58	[61, 63]
Propylone	No data	235.13	**236**, 188, 146, 218, 175, 160, 118	**86**, 149, 135, 121, 44	[61]
6-Methoxy-bk-MDMA	No data	237.11	**238**, 190, 175, 58	**58**, 204, 179	[61]
α-PHP	252, 251	245.36	**246**, 228, 175	**140**, 141, 105, 96, 77	[53, 57]
α-PiHP	No data	245.19	**246**, 140, 91, 119	**140**, 188, 98, 84	[61]
α-PHPP, PV8	253	259.39	**260**	**154**, 105, 77	[55]
α-POP, PV9	253	273.41	**274**	**168**, 105, 77	[55]
4-F-α-PVP	256	249.32	**250**	**126**, 95	[55]
4-F-α-PHP	No data	263.18	**264**, 140, 123, 109, 190, 137	**140**, 123, 96, 84, 69	[61]
4-F-α-PHPP	255	277.38	**278**	**154**, 123, 95	[57]
4-F-α-PV9, 4-F-α-POP	254, 253	291.40	**292**, 274, 221, 203, 189	**168**, 169, 123, 110, 95, 84, 55	[53]
4-Cl-α-PPP	263	237.10	**238**, 167, 185, 139, 98	**98**, 56, 111, 69	[62]
4-Cl-α-PHP	No data	279.15	**280**, 125, 140, 138, 209	**140**, 111, 84	[61]
4-Br-α-PVP	267	309.08	**310**/**312**, 160, 126, 168, 131, 183	**126**, 183, 155, 84	[62]
4-Methoxy-α-PVP	292	261.36	**262**	**126**, 135, 107	[56]
4-Methoxy-α-PHPP	292	289.41	**290**	**154**, 135	[57]
4-Methoxy-α-POP	292	303.44	**304**	**168**, 135	[57]
3,4-Dimethoxy-α-PVP	286, 316	291.39	**292**	**126**, 137, 165	[55]
Thiothinone	No data	169.24	**170**	**58**, 83, 111	[54]
α-PBT	No data	223.33	**224**	**112**	[60]
5-Br-α-PBT	No data	302.23	**302/304**	**112**	[60]
4-Br-α-PBT			**302/304**	**112**	
3-Br-α-PBT			**302/304**	**112**	
5-Br-α-PVT	No data	316.26	**316/318**	**126**, 189, 191	[60]
4-Br-α-PVT			**316/318**	**126**, 189, 191	
3-Br-α-PVT			**316/318**	**126**, 189, 191	
4,5-Br-α-PVT		395.15	**393/395**	**126**, 267, 269, 271	

The boldface figures shown in the data for LC–ESI-MS/MS and GC–EI-MS are precursor ions and base peaks, respectively

## Conclusions

Over the past 3 years, synthetic cathinones (along with synthetic cannabinoids) found in commercialized products and biological samples have been the most frequently identified group of designer drugs. Legislative efforts undertaken in many countries, including Poland, tend to eliminate them from the legal drug markets by adding them to lists of forbidden substances. However, the laboratories which produce novel psychoactive substances do not undergo toxicological or pharmacological control and can thus easily circumvent the law by freely introducing new derivatives which do not appear on the lists of forbidden substances. From the structures of synthetic cathinones that have been synthesized in the past 3 years and discussed in this review, it is clear that structural modification of the cathinone skeleton is virtually limitless. The structural diversity of already-synthesized cathinone derivatives encourages further modifications, mainly through the introduction of novel alkyl, alkoxy, or halogen substituents to the aromatic ring, and by playing with the length of the alkyl chain at the α-carbon atom. From the casualties reportedly caused by synthetic cathinones, it is clear that young people are the most vulnerable population group, as they are apt to experiment with novel designer drugs. In view of the imaginative and dynamic progress with respect to the synthesis of novel cathinone derivatives and the resulting adverse health effects and mortality, the generic scheduling of possession and/or use of substances which include a synthetic cathinone and its structurally modified derivatives seems to be the only legal remedy. For the time being, the identification and physicochemical characterization of novel synthetic cathinones constantly emerging on the designer drug market pose a considerable challenge for analytical chemists. Supplementing the existing databases with novel findings can significantly facilitate the efforts of toxicologists.
